# Sex differences in susceptibility to influenza A virus infection depend on host genotype

**DOI:** 10.1371/journal.pone.0273050

**Published:** 2022-09-16

**Authors:** Bristy Sabikunnahar, Karolyn G. Lahue, Loredana Asarian, Qian Fang, Mahalia M. McGill, Laura Haynes, Cory Teuscher, Dimitry N. Krementsov

**Affiliations:** 1 Department of Biomedical and Health Sciences, University of Vermont, Burlington, VT, United States of America; 2 Department of Immunology, University of Connecticut School of Medicine, UConn Center on Aging, Farmington, CT, United States of America; 3 Department of Medicine, University of Vermont, Burlington, VT, United States of America; 4 Department of Pathology, University of Vermont, Burlington, VT, United States of America; University of California San Diego, UNITED STATES

## Abstract

Infection with the respiratory pathogen influenza A virus (IAV) causes significant morbidity and mortality each year. While host genotype is thought to contribute to severity of disease, naturally occurring genetic determinants remain mostly unknown. Moreover, more severe disease is seen in women compared with men, but genetic mechanisms underlying this sex difference remain obscure. Here, using IAV infection in a mouse model of naturally selected genetic diversity, namely C57BL6/J (B6) mice carrying chromosomes (Chr) derived from the wild-derived and genetically divergent PWD/PhJ (PWD) mouse strain (B6.Chr^PWD^ consomic mice), we examined the effects of genotype and sex on severity of IAV-induced disease. Compared with B6, parental PWD mice were completely protected from IAV-induced disease, a phenotype that was fully recapitulated in the B6.Chr16^PWD^ strain carrying the PWD-derived allele of *Mx1*. In contrast, several other consomic strains, including B6.Chr3^PWD^ and B6.Chr5^PWD^, demonstrated greatly increased susceptibility. Notably, B6.Chr5^PWD^ and B6.ChrX.3^PWD^ strains, the latter carrying the distal one-third of ChrX from PWD, exhibited increased morbidity and mortality specifically in male but not female mice. Follow up analyses focused on B6 and B6.ChrX.3^PWD^ strains demonstrated moderately elevated viral load in B6.ChrX3^PWD^ male, but not female mice. Transcriptional profiling demonstrated genotype- and sex-specific gene expression profiles in the infected lung, with male B6.ChrX.3 mice exhibiting the most significant changes, including upregulation of a proinflammatory gene expression program associated with myeloid cells, and altered sex-biased expression of several X-linked genes that represent positional candidates, including *Tlr13* and *Slc25a53*. Taken together, our results identify novel loci on autosomes and the X chromosome regulating IAV susceptibility and demonstrate that sex differences in IAV susceptibility are genotype-dependent, suggesting that future genetic association studies need to consider sex as a covariate.

## Introduction

Seasonal infection with influenza A virus (IAV) is a significant threat to human health. IAV infects ~15% of the world’s population annually, resulting in ~1 million deaths each year [1–3]. Throughout history, multiple pandemics (three per century for the last 300 years) confirm the importance of IAV and, in the 20th century, three pandemics in particular have been exceptionally devastating [1–3].

The incidence, severity, and fatality rate associated with IAV are thought to be a function of viral pathogenicity [4, 5] and/or immunity due to previous exposure [6]. Consequently, extensive research has investigated IAV evolution, virulence, and protective immunity [7, 8]; however, little is known about host genetic factors in disease susceptibility and severity (reviewed in [9]). Most people who get IAV recover in less than 2 weeks. However, a number of infected patients become critically ill and require intensive care. A subset rapidly develop severe progressive respiratory failure that is often associated with multi-organ failure and/or marked worsening of underlying airways disease and death [10]. Epidemiological studies have shown that more than 90% of human H5N1 infections have occurred in extended genetically related family members, suggesting a possible role for host genetics [11], and a study of IAV-associated deaths in Utah over the past 100 years provided strong evidence of host genetics as a risk factor [12]. Notwithstanding, population-based human genetic studies have not yet uncovered common alleles with a significant impact on IAV susceptibility (reviewed in [9]). Nonetheless, more recent whole genome sequencing-based studies in humans have identified rare loss-of-function allelic variants at four different genes in monogenic life-threatening IAV pneumonia: *GATA2*, *TLR3*, *IRF7*, and *IRF9* (reviewed in [13]). While serving as an important proof-of-principle, these rare alleles fail to explain the majority of the natural genetic variation underlying the observed large inter-individual variability in IAV susceptibility, and thus animal studies are needed.

Epidemiological evidence from both seasonal outbreaks of IAV and pandemics suggests that morbidity and mortality are significantly greater in women than in men [14]. For example, a 2010 report from the WHO concluded that the outcome of pandemic IAV, as well as avian H5N1, was generally worse in young adult females [15]. However, in epidemiological studies where age and sex were included as covariates, the data indicate that incidence and severity change as a function of age, with more males being affected from birth through 15 years of age, and more females affected across all post-pubertal age ranges [1–3, 16–24].

Studies in mice have, in general, confirmed that adult males are more resistant to IAV than females, and have significantly contributed to characterizing sex differences in IAV pathogenesis [25, 26]. Based on animal experimentation, the sex difference in IAV infection is believed to be primarily a function of the cell-extrinsic immunostimulatory effects of estrogens and immunosuppressive effects of testosterone on the immune system [27, 28]. Interestingly, females who typically generate higher innate and adaptive immune responses due to the immune enhancing effects of estrogens should be able to accelerate virus clearance and reduce virus load more readily than males. As such, morbidity and mortality should be less in females compared to males; however, epidemiology suggests the opposite is true (see above). The disparity between the epidemiological data and the immunostimulatory effects of estrogens, and suppressive effects of testosterone, are therefore attributed to a sex difference in immunopathology, with female lung function being more severely impacted through the damaging effects of leukocytes on epithelial and endothelial cells [26].

Beyond the role of hormones, the role of sex chromosomes in sex differences in IAV susceptibility has emerged. While our previous studies demonstrated minimal effects of the sex chromosome complement (XX vs. XY) *per se* [25], our more recent findings established that polymorphic variants on the Y chromosome can significantly impact IAV susceptibility and associated inflammatory responses in the lung in male mice [29].

A key limitation to animal studies on sex differences in IAV pathogenesis is that most of them have performed in a single commonly used laboratory strain of mice, C57BL/6 (B6). Given the enormous genetic diversity in human populations, this is provides a very narrow view, representing a single artificially selected genotype. These limitations can be overcome by incorporating into the experimental design so-called wild-derived inbred mouse strains, thereby more accurately modeling the greater evolutionarily selected genetic diversity seen in humans. One such wild-derived inbred strain is the PWD/PhJ (PWD) strain [30]. We have leveraged this model, together with the tractable chromosome (Chr) substitution (consomic) strains, which carry PWD chromosomes (one at a time) on the B6 background (denoted B6.Chr^PWD^), to map the genetic basis of several immune mediated diseases [31–35].

Here, we used a well-established model of severe infection with the mouse-adapted IAV H1N1 strain in the classic laboratory strain B6 compared with the wild-derived PWD strain, and found that the PWD mice are completely resistant to IAV infection. We next used the full panel of available B6.Chr^PWD^ consomic mice to map susceptibility and resistance loci. Our findings demonstrate that while wild-derived PWD alleles on Chr16 are sufficient to confer complete protection from IAV challenge, PWD alleles on other chromosomes impart enhanced susceptibility. Importantly, we demonstrate that in several cases such genotype-determined susceptibility is sex-dependent. This is exemplified by enhanced susceptibility of male mice carrying PWD-derived alleles on distal ChrX, which is associated with elevated virus replication and dysregulated sex-dependent inflammatory gene expression in the lung. Taken together, our findings suggest that sex differences in IAV severity are highly dependent on genotype.

## Materials and methods

### Animals

C57BL/6J (B6), PWD/PhJ (PWD), and B6.Chr^PWD^ consomic mice were purchased from Jackson Laboratories (Bar Harbor, ME, USA), then bred and housed in a single room within the animal facility at the University of Vermont (UVM) for five or more generations. B6.Chr^PWD^ consomic mice were subjected to genome-wide SNP genotyping using DartMouse genotyping services (Dartmouth College, NH, USA), to confirm their correct genotypes and to enhance rigor and reproducibility of these studies, as previously described by us [35, 36]. All mice used in this study were of the expected genotypes, with the following exception, previously described [35]. B6.Chr17S^PWD^ mice were found to carry a homozygous B6-derived interval between 30 and 45 Mb on Chr17, encompassing *H-2*. B6.Chr17F^PWD^ mice carrying the fully PWD-derived Chr17 were generously provided by Dr. Jiri Forejt (Institute of Molecular Genetics of the ASCR, Czech Republic) and housed in the vivarium at UVM for two or more generations prior to experimentation. The experimental procedures used in this study were approved by the Animal Care and Use Committee of the University of Vermont.

### Viruses and infections

Mouse-adapted Puerto Rico A/PR/8/34 H1N1 (PR8) influenza A virus (IAV) was obtained from Charles River Laboratories. *In vivo* infectious dose was determined empirically by *in vivo* titration of the stock virus in B6 mice. For virus inoculation, mice were lightly anesthetized using isoflurane and respective doses of PR8 IAV (as described in Results) were administered intranasally in 0.05 ml of PBS. In the consomic genetic screen study, to eliminate initial sex differences, all female mice received a 33% lower dose of IAV compared with males. Mice were monitored daily for weight loss and other clinical signs of illness. Animals that became grossly moribund were euthanized.

### Anti-viral titer measurement by ELISA

Serum was collected from PWD mice on day 22. Anti-IAV antibody titer was measured using ELISA against immobilized PR8 IAV virus, as previously described [37]. Endpoint serum titers were defined by the last serum dilution that gave an OD reading that was 2 standard deviations above the mean of negative control OD.

### Whole lung RNA analysis

Mice were euthanized by CO_2_ inhalation. Whole lungs were dissected and placed into tubes containing 2.3 mm silica beads (BioSpec Products, OK, USA) and Trizol reagent (Invitrogen, CA, USA). Whole lung tissues were homogenized using a BeadBeater homogenizer (BioSpec Products, OK, USA). RNA was extracted using Direct-zol RNA Microprep Kit (Zymo Research, CA, USA) according to manufacturer protocol. RNA concentration was determined by Nanodrop (Thermo Scientific NanoDrop 2000 Spectrophotometer). Influenza viral load was determined by measuring the expression of viral gene NS1 by qRT-PCR [38]. For this purpose, cDNA was synthesized from the RNA isolated from lung, using qScript® cDNA Synthesis Kit (Quantabio, USA). The qPCR reaction was performed according to the manufacturer’s instructions using the DyNAmo ColorFlash SYBR Green kit by Thermo Fisher Scientific (Waltham, MA), using specific primers. Target gene expression was normalized by the expression of the housekeeping gene *B2m* (β-2-Microglobulin) and calculated by a comparative Ct method formula 2^-(deltaCt), and multiplied by a factor of 10,000 for ease of visualization, i.e. 2^-(Ct^target gene^–Ct^*B2m*^)*10,000.

### RNAseq and bioinformatic analyses

Transcriptional profiling of PR8-infected lungs was done by RNAseq. RNA was extracted as described above, followed by quantification, quality assessment, library preparation, and sequencing at the Vermont Integrative Genomics Resource core facility at UVM. cDNA libraries were synthesized using SMARTer Stranded Total RNA-Seq Kit v2—Pico Input Mammalian (Takara, Japan), according to manufacturer protocol. Ribosomal RNA depletion was done to ensure recovery of a range of RNA species. Single-end 75 bp sequencing was performed on an Illumina HiSeq1500, and ~30 million reads per sample were generated. Demultiplexed sequencing data was received from the sequencing facilities and quality control of the sequencing data was done using FastQC platform. Genomic alignment and transcript quantification from RNA-seq data was performed using Salmon tool [39]. All raw and processed RNAseq data have been deposited in the GEO repository under accession number GSE210905. Differential gene-expression analyses were conducted using DEseq2 in R studio [40]. Cutoffs for differential gene expression are described in the Results section and figure and table legends. Gene ontology (GO) pathway enrichment analysis on differentially expressed genes was performed using PANTHER [41]. Cell type enrichment analysis was performed using the ImmGen website [42], using the My Geneset tool, using the ImmGen ULI RNAseq reference dataset and select reference cell populations of interest.

### Statistical analyses

Statistical analyses not pertaining to RNA-seq data were carried out using GraphPad Prism software version 8.4.3 (GraphPad Software, CA, USA). The specific tests used to assess the significance of the observed differences are detailed in the corresponding figure legends. All center values represent the mean, and error bars represent the standard error of the mean. A P-value of ≤0.05 was considered significant.

## Results

### PWD-derived alleles on Chr16 are highly protective against IAV challenge

**Fig 1:**
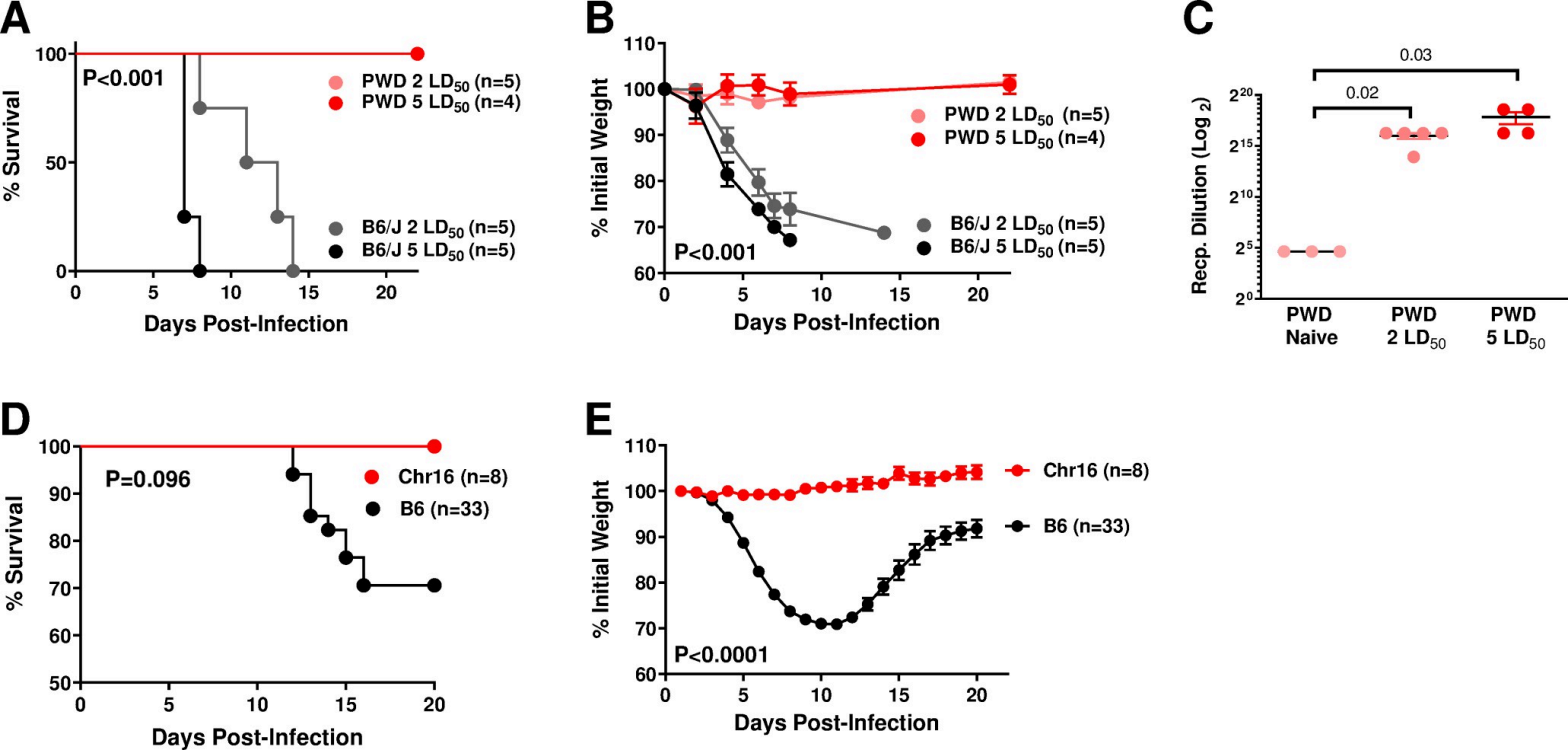
PWD alleles on Chr16 provide complete protection from lethal IAV challenge. (**A–C**) Male B6 and PWD mice were challenged with 2 LD_50_ or 5 LD_50_ doses of PR8 IAV (LD_50_ dose was pre-determined on B6 mice). Survival (**A**) and weight loss (**B**) are shown, with P values in each panel indicating overall significance of differences between the groups, as determined by Mantel-Cox test and 2-way ANOVA, respectively. (**C**) On day 22 post-infection, serum was collected from PWD mice and uninfected naïve PWD mice, and anti-IAV antibody titers were determined by ELISA (as described in the Materials and Methods). P values indicate the significance of difference between the indicated groups, as determined by student’s T test. (**D, E**) Male and female B6 and B6.Chr16^PWD^ (Chr16) mice were challenged with ~LD_50_ of PR8 IAV. Survival (**D**) and weight loss (**E**) are shown, representing pooled data from males and females with P values in each panel indicating significance of differences between the strains, as determined by Mantel-Cox test and 2-way ANOVA, respectively. The number of animals per condition is indicated in each panel.

In order to begin to identify genetic variants driving differential IAV susceptibility, we intranasally challenged B6 and PWD mice with 2 or 5 ˣ 50% lethal dose (LD_50_; determined empirically by preliminary titration experiments on B6 mice) of mouse-adapted A/Puerto Rico/8/1934 (PR8) H1N1 IAV. While B6 mice rapidly succumbed to infection as expected, PWD mice were completely protected from mortality and infection-induced weight loss (**Fig 1A** and **1B**). In spite of a lack of symptomatic infection, PWD mice mounted robust anti-viral antibody responses (**Fig 1C**), confirming successful infection.

It is well established that most common laboratory inbred strains of mice, such as B6, carry a non-functional allele of *Mx1*, a key IAV resistance gene [43]. In contrast, most wild-derived mouse strains carry a functional protective allele of *Mx1*, which renders them highly resistant to IAV challenge [43]. Because *Mx1* resides on mouse Chr16, we assessed the IAV susceptibility of B6.Chr16^PWD^ consomic mice. Compared with B6 controls, B6.Chr16^PWD^ mice were completely protected from mortality and weight loss induced by IAV infection (**Fig 1D** and **1E**). These results demonstrate that PWD-derived alleles on Chr16 are sufficient to provide complete resistance to IAV, although they do not rule out the possibility of the existence of additional protective alleles on Chr16 besides *Mx1*. The finding that Chr16^PWD^ is sufficient to recapitulate the IAV resistance of parental PWD mice raises the question whether additional alleles regulating IAV susceptibility exist elsewhere in the PWD genome.

### PWD alleles exert bidirectional effects on susceptibility to IAV infection

**Fig 2:**
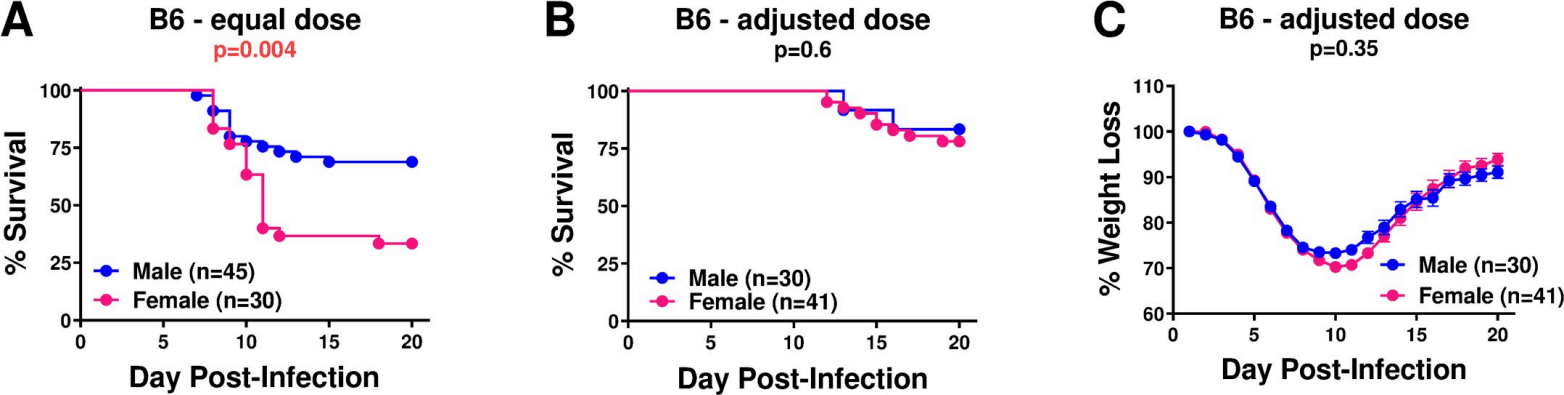
Sex differences and normalization of IAV severity in male and female B6 mice. (**A**) B6 male and female mice were challenged with equal doses of PR8 IAV, and survival was assessed. (**B** and **C**) B6 male and female mice were challenged with sex-adjusted estimated LD_50_ of PR8 IAV (33% lower dose for females). Significance of differences in survival between females and males was determined using the Mantel-Cox test, significance of differences in weight loss was determined by two-way ANOVA (overall effect of sex). P values are indicated below each panel title. The number of animals per condition is indicated in each panel.

In order to identify additional PWD loci regulating IAV severity, we performed a genome-wide screen in B6.Chr^PWD^ consomic and sub-consomic mice, including sex as a key biological variable. Female B6 mice are reported to exhibit higher mortality compared with male B6 mice when infected at the same dose [14, 29], thus mirroring increased susceptibility seen in adult human females [19]. Indeed, in our hands, male and female B6 mice infected with equal doses of PR8 IAV demonstrated a similar outcome (**Fig 2A**). Thus, in order to achieve maximum statistical power in our genetic screen, we decreased the female dose by 33% to reach similar survival and weight loss for both sexes in B6 mice (**Fig 2B** and **2C**), allowing us to pool male and female data for each strain. These sex-adjusted doses were used for the remainder of our experiments. We note that, in B6 mice, these adjusted doses were closer to LD_20_ rather than the originally intended LD_50_.

**Fig 3:**
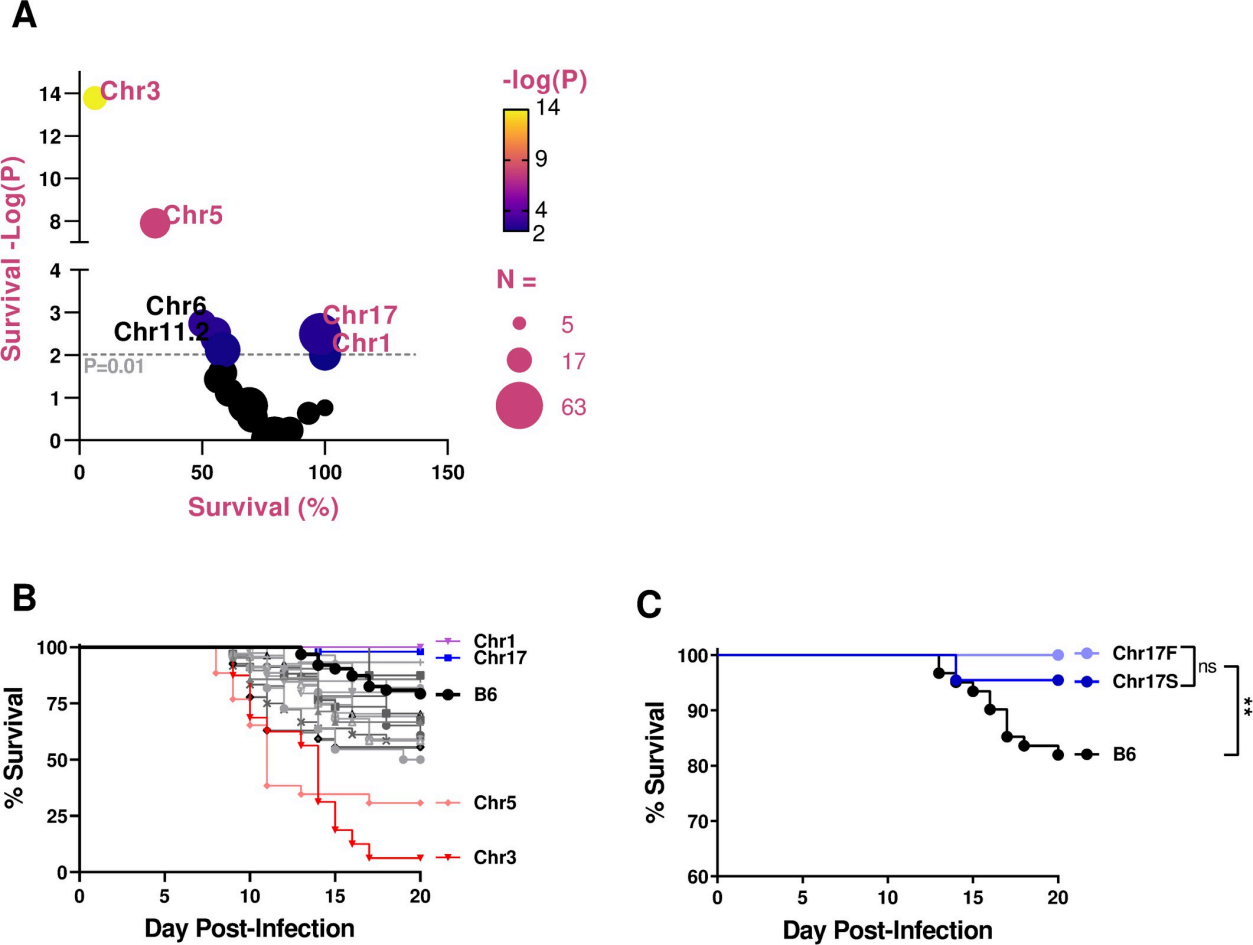
IAV challenge of B6.Chr^PWD^ consomic mice reveals a wide range of susceptibility phenotypes. B6 control and B6.Chr^PWD^ male and female mice were infected with sex-adjusted doses of PR8 as in **Fig 2B**. Significance of difference in survival between B6 and each consomic strain (male and female data pooled by strain) was assessed by Mantel-Cox test. (**A**) A bubble volcano plot showing significance of survival (-Log_10_(P)) compared with B6 plotted vs. % survival, across all consomic strains, with strains of interest labeled. The size of the circle indicates the total number of animals studied per strain (N), as indicated. (**B**) Survival analysis of B6 and consomic mice, with strains of interest labeled (male and female data pooled by strain). Complete data on survival analysis for each strain are presented in **Table 1**. (**C**) Comparison of survival between B6 and B6.Chr17^PWD^ consomics, Chr17S (short) and Chr17F (full), (male and female data pooled by strain). ** signifies a P value of <0.01, as determined by Mantel-Cox test.

Female and male B6 and B6.Chr^PWD^ mice were challenged with the sex-adjusted dose of PR8 as above, and survival was assessed using pooled data from females and males for each strain. Because of the large number of strains studied, multiple independent cohorts of mice were used, including B6 controls infected in parallel with each cohort of consomic strains. The number of individual mice studied was largely determined by breeding performance and availability (numbers for each strain and sex are indicated in **Table 1**). B6.Chr^PWD^ consomic mice exhibited a broad range of susceptibility to IAV, with two strains demonstrating markedly enhanced susceptibility: B6.Chr3^PWD^ and B6.Chr5^PWD^ (**Fig 3A and 3B** and **Table 1**). Several strains exhibited significantly higher survival compared with B6, namely B6.Chr17^PWD^, and B6.Chr1^PWD^, **Fig 3A and 3B** and **Table 1**. Because it carries the murine major histocompatibility (MHC) gene complex, a.k.a. the *H-2*, Chr17 is of particular interest. To address its role, we used two different strains, B6.Chr17F^PWD^, carrying the full length Chr17 derived from PWD, and B6.Chr17S^PWD^, which carries a B6-derived locus encompassing the MHC in the middle of an otherwise PWD-derived Chr17, described in our previous studies [35]. Interestingly, both strains exhibited similar resistance to IAV infection compared with B6 (**Fig 3C**), suggesting that PWD-derived alleles on Chr17 outside of the MHC locus regulate susceptibility to IAV infection. Taken together, our results demonstrate that PWD alleles on several chromosomes exert potent bidirectional effects on susceptibility to IAV infection.

**Table 1:** Survival analysis of IAV-challenged consomic strains using pooled sex data. The indicated number (N) of each strain were challenged with IAV as described in **Fig 3**. Male and female data were pooled. Significance of differences in survival between B6 and each consomic strain were assessed using the Mantel-Cox test and the P value are reported, with P<0.05 shown in bold font. Percent of mice surviving to the endpoint on day 20 is shown. Numbers of male and female mice studied are also shown. Data from for B6.Chr17^PWD^ represent pooled data from Chr17S and Chr17F strains. The low N for some strains (Chr13, Chr7, etc.) was a result of low availability of animals due to poor breeding performance.

Strain	Survival (P)	% Survived	N =	Male:Female
B6	N/A	79.4	63	24:39
Chr1	**0.0098**	**100.0**	29	14:15
Chr2	0.15	66.7	21	13:8
Chr3	**<0.0001**	**6.3**	16	9:7
Chr5	**<0.0001**	**30.8**	26	16:10
Chr6	**0.002**	**50.0**	22	10:12
Chr7	0.57	87.5	8	4:4
Chr9	**0.03**	**58.3**	24	13:11
Chr10.2	0.94	80.0	20	10:10
Chr10.3	**0.004**	**55.6**	27	12:15
Chr11.1	0.88	80.0	35	19:16
Chr11.2	**0.003**	**55.2**	29	15:14
Chr11.3	0.23	93.3	15	5:10
Chr12	**0.008**	**58.3**	36	22:14
Chr13	0.99	80.0	5	3:2
Chr14	0.15	67.6	34	17:17
Chr15	**0.04**	**56.5**	23	11:12
Chr17	**0.003**	**98.0**	50	30:20
Chr18	0.29	70.4	27	14:13
Chr19	0.15	69.2	39	27:12
ChrX.1	0.59	85.7	21	6:15
ChrX.2	0.17	100.0	8	5:3
ChrX.3	0.08	60.9	23	10:13
mt	0.76	82.1	28	11:17

### Genetic variation regulates sex differences in susceptibility to IAV infection

**Fig 4:**
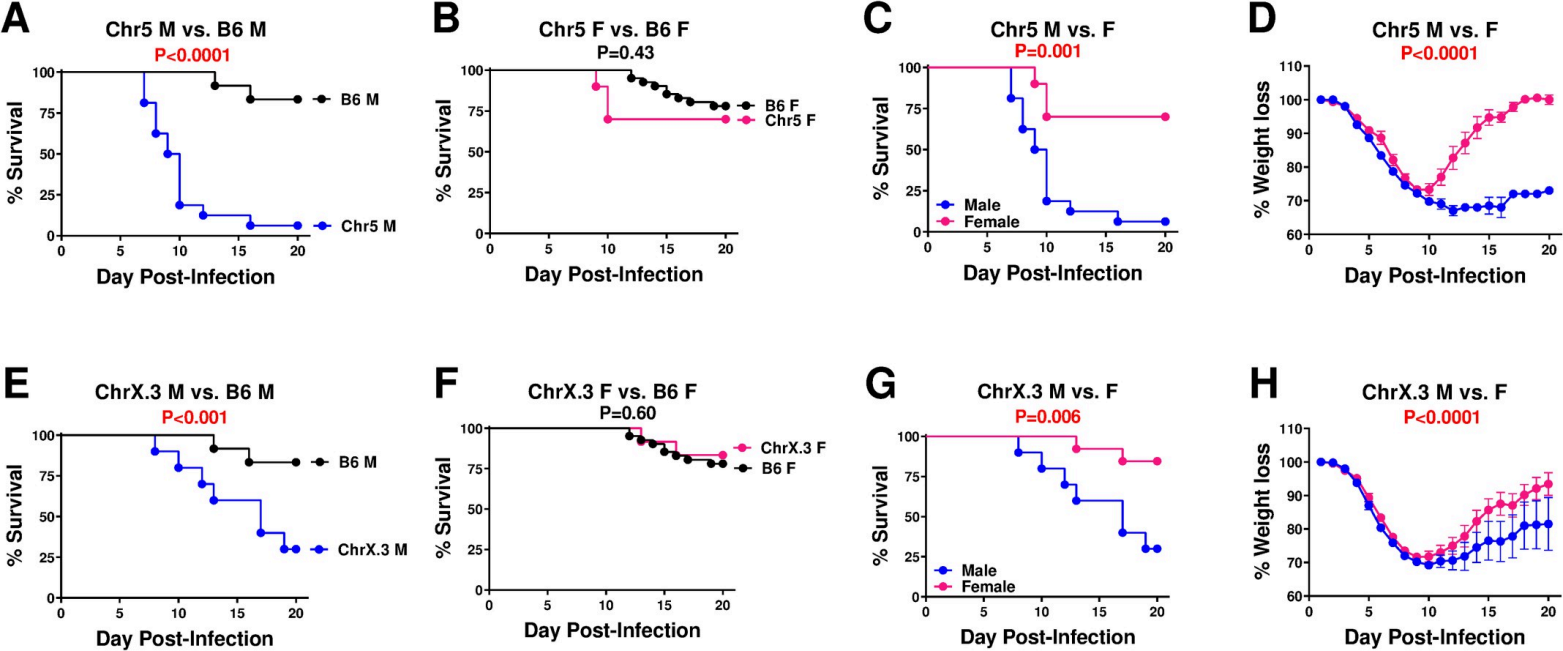
Genotype-dependent sex differences in IAV susceptibility of B6.Chr^PWD^ consomic mice. Male (M) and female (F) B6 control and B6.Chr5^PWD^ (**A-D**) and B6.ChrX.3^PWD^ (**E-H**) mice were infected with sex-adjusted doses of PR8 as in **Fig 2B**. The following comparisons were made: (**A** and **E**) male consomic vs. male B6 control, (**B** and **F**) female consomic vs. female B6 control, and (**C, D, G** and **H**) male vs. female of each consomic. Significance of difference in survival for each comparison assessed by Mantel-Cox test, while significance of difference in weight loss was assessed by two-way ANOVA (overall effect of sex). The P value for each comparison is indicated in each corresponding graph, in red font if below P<0.05. Complete data on survival analysis for all consomic strains are presented in **Tables 1** and 2.

Although with our sex-normalized dose of IAV, male and female B6 mice did not show significant differences from one another (**Fig 2B** and **2C**), we performed sex-disaggregated analyses for each strain to identify any potential sex-by-genotype interactions. To this end, comparisons were performed between males and females *within* each strain, as well as comparing each strain versus B6 controls within one sex at a time (**Table 2**). Two strains, B6.Chr5^PWD^ and B6.ChrX.3^PWD^ (the latter carrying the distal ~1/3 of ChrX derived from PWD), demonstrated a marked sex difference in both types of comparisons (**Table 2**), with males demonstrating significantly lower survival and increased weight loss compared with B6 males (**Fig 4A, 4B, 4E and 4F**) or compared with females of the same strain (**Fig 4C, 4D, 4G and 4H**). While several other strains (e.g., B6.Chr1^PWD^) showed significant differences from B6 in only one sex and not the other, they lacked a significant difference in a direct male to female comparison *within* strain (**Table 2**), and thus are unlikely to represent a true sex-by-genotype interaction. Taken together, these data suggest that PWD alleles on Chr5 and ChrX selectively increase IAV susceptibility in males and not females. Given the known role for the X chromosome in sex differences [28], we focused our subsequent studies on B6.ChrX.3^PWD^ mice (abbreviated as ChrX.3^PWD^).

**Table 2:** Survival analysis of IAV-challenged consomic strains using sex-stratified data. Male and female consomic mice were challenged with IAV as described in **Fig 3**. Significance of differences in survival assessed using the Mantel-Cox test and the P value are reported, with P<0.05 shown in bold font. The following three comparisons were made: male vs. females of each consomic strain (Male vs. Female, column 1), consomic males vs. B6 males (Consomic M vs. B6 M, column 2), and consomic females vs. B6 females (Consomic F vs. B6 F, column 3).

Strain	Male vs. Female	Consomic M vs. B6 M	Consomic F vs. B6 F
B6	0.54		
Chr1	>0.99	0.11	**0.049**
Chr2	0.15	**0.032**	0.57
Chr3	0.65	**<0.0001**	**<0.0001**
Chr5	**0.001**	**<0.0001**	0.48
Chr6	0.38	0.11	**0.004**
Chr7	0.32	0.39	0.98
Chr9	0.88	0.10	0.11
Chr10.2	0.27	0.31	0.40
Chr10.3	0.73	0.05	**0.03**
Chr11.1	0.32	0.34	0.45
Chr11.2	0.49	0.06	**0.014**
Chr11.3	0.16	0.77	0.11
Chr12	0.47	**0.016**	0.30
Chr13	0.41	0.44	0.47
Chr14	0.62	0.14	0.57
Chr15	0.62	**0.041**	0.22
Chr17	0.22	**0.021**	0.09
Chr18	0.53	0.15	0.91
Chr19	0.57	0.12	0.81
ChrX.1	0.90	0.99	0.51
ChrX.2	>0.99	0.34	0.38
ChrX.3	**0.006**	**0.001**	0.55
mt	0.051	0.21	0.14

### Genetic variation regulates sex differences in viral replication and host response during IAV infection

**Fig 5:**
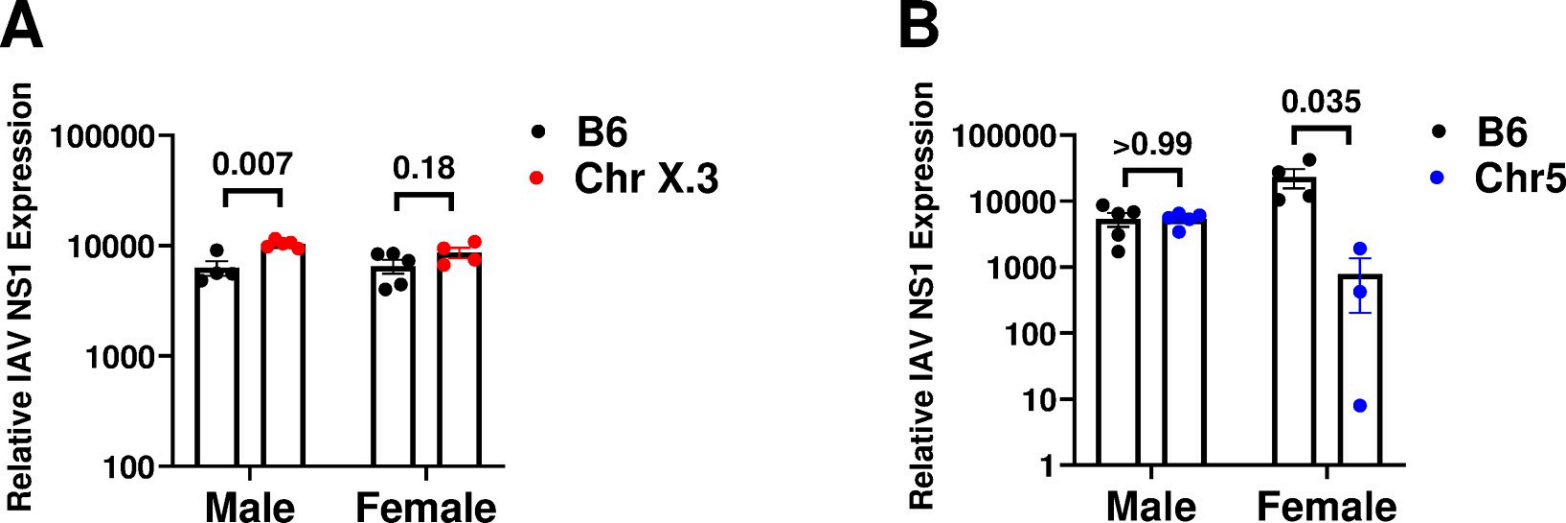
Differences in IAV viral load between B6, ChrX.3^PWD^, Chr5^PWD^ and male and female mice. Male and female B6 and ChrX.3 mice (**A**) or B6 and Chr5 mice (**B**) were infected with PR8 IAV as in **Fig 3**. On day 6 post-infection, whole lung RNA was extracted as described in the Materials and Methods. Relative expression of IAV NS1 gene RNA was assessed using qRT-PCR and normalized to host housekeeping gene *B2m*. Data were analyzed by two-way ANOVA with Sidak’s post-hoc comparisons, with P values representing significance of differences as indicated for each comparison.

Susceptibility to severe IAV-induced disease could be driven by the inability of the host to control viral replication and cytopathic effects. Alternatively, it could be driven by collateral damage due to host inflammatory responses. To determine the molecular mechanism(s) underlying sex-specific genetic regulation of IAV susceptibility, we examined viral replication and host gene expression in the lung during infection at day 6 post challenge, just prior to onset of mortality in B6.ChrX.3^PWD^ mice (**Fig 4E**). Male and female B6 and B6.ChrX.3^PWD^ mice were challenged with IAV as above, followed by isolation of RNA from whole lung tissue. Viral replication was assessed using qRT-PCR analysis to measure expression of the viral NS1 gene RNA as a surrogate for viral load. We found that viral load was significantly higher levels in male B6.ChrX.3^PWD^ mice compared with male B6 mice, a difference that in females followed a similar trend, but was of a lower magnitude and did not reach significance (**Fig 5A**). These data suggest moderately higher viral loads in male B6.ChrX.3^PWD^ hosts. In separate experiments, we compared viral loads in B6.Chr5^PWD^ mice to B6 mice, since this strain also demonstrated a sex difference in IAV severity. Surprisingly, we found comparable viral loads between male B6.Chr5^PWD^ and male B6 mice, while the viral load in B6.Chr5^PWD^ females was lower than that of B6 females. These results suggest that the sex difference in IAV severity in B6.Chr5^PWD^ mice may be driven by superior virus control in females, and likely involving a distinct mechanism from that in B6.ChrX.3^PWD^ mice.

**Fig 6:**
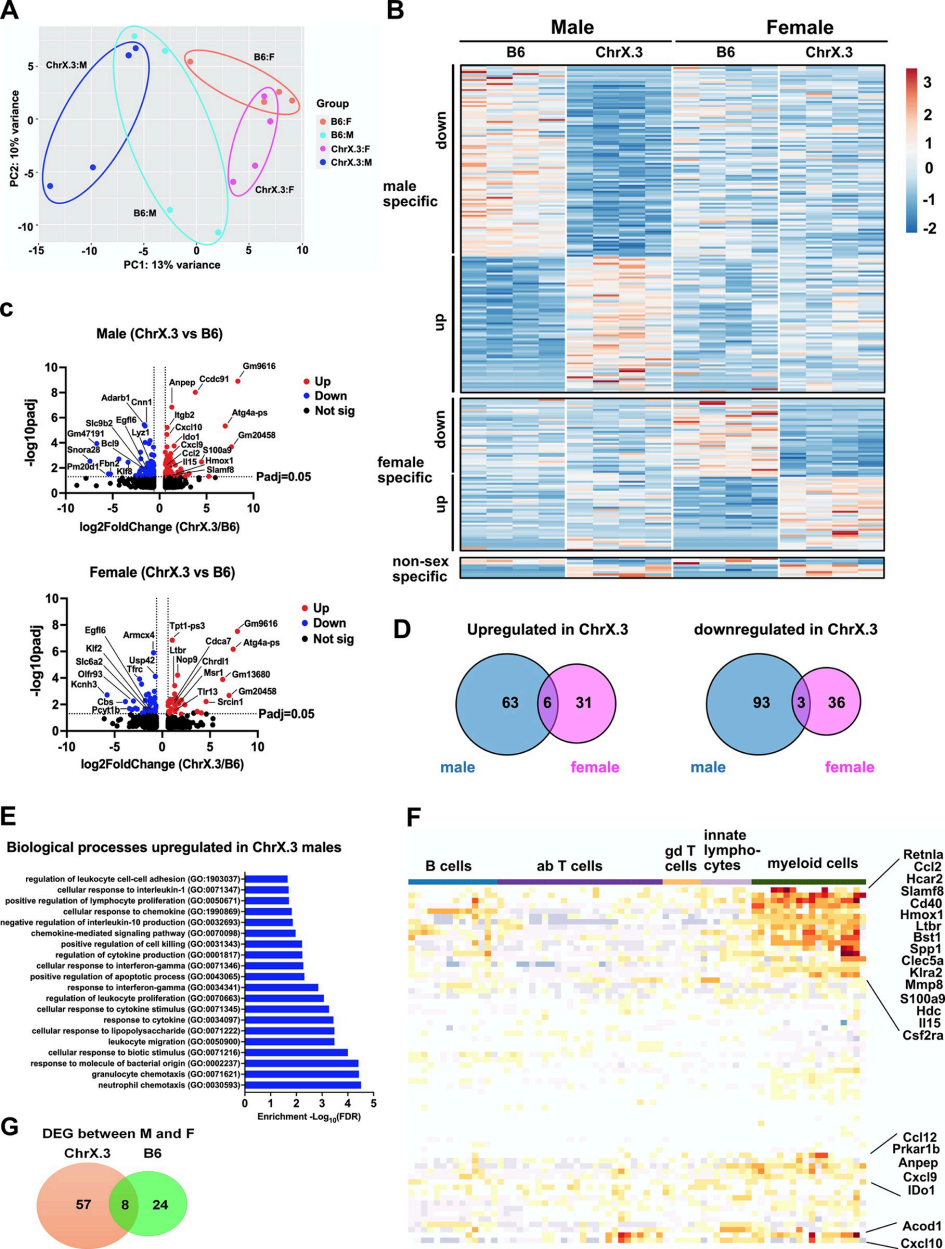
Sex differences in genetically regulated host lung responses in B6 and ChrX.3^PWD^ mice. Male and female B6 and B6.ChrX.3^PWD^ (ChrX.3) mice were infected with PR8 IAV as in **Fig 3**. On day 6 post-infection, whole lung RNA was processed for RNA sequencing and analysis as described in Materials and Methods. (**A**) Principal component analysis was performed in DEseq2, demonstrating clustering of individual samples by sex and genotype, as annotated. (**B, C,** and **D**) DEseq2 was used to determine differentially expressed genes between X.3 and B6 mice within males or females, as annotated, using a cutoff of |Log2 (Fold Change)|>0.6 and P_adjusted_ (P_adj_) <0.05. (**B**) A row-normalized gene expression heatmap showing DEGs in males, females, or both sexes, as indicated. Volcano plots (**C**) and Venn diagrams (**D**) illustrating differential gene expression between ChrX.3 and B6. (**E**) Biological pathway enrichment analysis on DEGs upregulated in ChrX.3 in males was performed using Gene Ontology as described in Materials and Methods. Top 20 enriched pathways are shown. (**F**) ImmGen MyGeneSet analysis was performed on DEGs upregulated in ChrX.3 in males as described in the Materials and Methods. Heatmap indicates relative expression of DEGs in cell populations of interest, with each column representing a cell type or cells state within the broader cell categories labeled across the top. (**G**) A Venn diagram indicating overlap between genes differentially expressed between males vs. females in ChrX.3 and B6 mice.

To determine the role of genetic variation of host during IAV infection on gene expression in the infected lung, we performed RNA sequencing (RNAseq) analysis on whole lung RNA isolated from male and female B6 and B6.ChrX.3^PWD^ mice on day 6 post-infection with IAV. Principal component analysis demonstrated distinct sample clustering by sex and genotype (**Fig 6A**). To assess the impact of genotype on the lung transcriptional profile in each sex, we performed differential gene expression analyses comparing B6.ChrX.3^PWD^ versus B6 in each sex. This analysis revealed that more genes were differentially expressed between ChrX.3 and B6 in males compared with females (165 vs 76 genes, respectively; **Fig 6B, 6C; S1 File, A** and **B**). More strikingly, B6.ChrX.3^PWD^ genotype-specific differentially expressed genes (DEGs) exhibited minimal overlap between male and females (**Fig 6B and 6D**). To identify molecular pathways associated with these distinct genotype- and sex-dependent transcriptional profiles, we performed enrichment analysis using Gene Ontology (GO) on sets of DEGs upregulated or downregulated in ChrX.3 in each sex. The gene set that showed the most significant pathway enrichment was the DEGs upregulated in ChrX.3 males (**Fig 6E**). The biological processes that were associated with DEGs upregulated in ChrX.3 males included many major immune response pathways including chemotaxis, leukocyte migration, cytokine response and regulation, cellular response to interferon gamma, and cell death (**Fig 6E**), including key immune response genes *Cxcl9*, *Cxcl10*, *Ccl2*, *Il15*, *Slamf8* and *S100a9* (**Fig 6C**). To infer the contribution of lung infiltrating immune cells to these whole lung transcriptional profiles, we used ImmGen MyGeneset analysis, which revealed a distinct myeloid cell signature in a subset of our DEGs of interest (**Fig 6F**). Taken together, our results reveal that the ChrX.3^PWD^ genotype drives a distinct sex-specific gene expression profile in the infected lung, that is more pronounced in males, and consistent with and enhanced inflammatory state driven by myeloid cells.

A possible mechanism by which the ChrX.3^PWD^ locus could regulate IAV susceptibility would be through polymorphisms driving differential expression of positional candidate genes encoded in this locus, in the infected lung. To address this possibility, we filtered the aforementioned DEG lists by location in the ChrX.3^PWD^ locus (88.4 Mb through distal end of ChrX). This identified 4 genes in males, 6 genes in females, and 2 in both sexes, that were differentially expressed in ChrX.3^PWD^ versus B6 IAV-infected lungs and mapped to the X.3^PWD^ locus (**Table 3**). With the notable exception of *Tlr13* [44], which was upregulated in X.3^PWD^ females, none of these genes had an obvious documented function in viral replication, immune response, or lung pathology.

**Table 3:** Positional candidate genotype-dependent DEGs that mapped to ChrX.3 interval. Genes differentially expressed between ChrX.3 males and B6 males (male), ChrX.3 females and B6 females (female), or in both sexes (both), which mapped to Chr X.3 interval (Chr = X; position = 88,400,000 bp or greater) from the RNAseq data described in **Fig 6**. Direction of change, up indicates increased expression in ChrX.3 relative to B6, down indicates decreased expression. A cutoff filter of |log2(FoldChange)| = 0.6, Padj = <0.05 was used to identify DEGs.

*Gene Symbol*	*Direction in ChrX*.*3*^*PWD*^	*Position on Chr X (bp)*		*Sex in which differentially expressed*
		Start	end	
*Klf8*	down	153,237,466	153,396,132	**male**
*Platr21*	down	139,240,226	139,357,738	**male**
*Capn6*	down	143,802,236	143,827,412	**male**
*Nexmif*	up	104,077,434	104,201,185	**male**
*Pcyt1b*	down	93,654,863	93,749,951	female
*Armcx4*	down	134,686,519	134,696,757	female
*Bex3*	down	136,270,253	136,271,978	female
*Tlr13*	up	106,143,275	106,160,493	female
*Chrdl1*	up	143,285,674	143,394,262	female
*Nlgn3*	up	101,299,179	101,321,350	female
*G530011O06Rik*	down	169,975,043	169,978,917	*both*
*Egfl6*	down	166,523,007	166,585,716	*both*

In parallel, to directly assess differences in gene expression between males and females across the two distinct genotypes, B6 and ChrX.3^PWD^, we identified sex-dependent DEGs in each strain. This identified 57 sex-dependent DEGs in ChrX.3^PWD^ mice, 24 in B6, and 8 in common (**Fig 6G; S1 File, C** and **D**). The 8 overlapping DEGs that were differentially expressed between males and females in both strains included the expected Y-encoded genes *Kdm5d*, *Uty*, *Ddx3y*, *Eif2s3y*, and the well-known X-linked female-biased genes *Xist*, *Kdm5c*, *Eif2s3x* (higher expression in females) (**Table 4**), as well as the *Tpt1-ps3* pseudogene on Chr6. The rest of the sex-dependent DEGs were specific either to ChrX.3^PWD^ or B6 (**Fig 6G**), suggesting that sex differences in gene expression in this model are highly genotype dependent. Analysis of the ChrX-linked DEGs identified 4 additional genes that were differentially expressed between males and females only in ChrX.3^PWD^ mice, including *Kdm6a*, *Gm2223*, *Bgn*, *Slc25a53*, and *Rps7-ps3* (**Table 4**). Of these, only the latter two genes map to the ChrX.3^PWD^ locus, representing positional candidates, while the others lie on the proximal end of ChrX. Taken together, our results suggest that enhanced severity of IAV infection in male B6.ChrX.3^PWD^ mice is accompanied by moderately enhanced viral load and dysregulated sex-biased inflammatory gene expression in the lung, and highlight several positional candidate genes of interest in the ChrX.3 locus to be investigated in future studies.

**Table 4:** Sex-dependent DEGs mapping to the sex chromosomes. Genes differentially expressed between males and females in: B6 mice only (B6; none in this category), ChrX.3 mice only (ChrX.3), or in both strains (both) which mapped to ChrX or ChrY, from the RNAseq data described in **Fig 6**. Direction of change: up indicates increased expression in males relative to females, down indicates increased expression in females relative to males. A cutoff filter of Padj = <0.05 was used to identify DEGs, without a fold change cutoff.

*Gene Symbol*	*Chr*	*Direction in males*	*Position on Chr (bp)*		*Strain in which differentially expressed*
			Start	end	
*Kdm6a*	X	down	18,162,575	18,279,936	ChrX.3
*Gm2223*	X	down	33,505,661	33,507,010	ChrX.3
*Bgn*	X	down	73,483,602	73,495,933	ChrX.3
*Eif2s3x*	X	down	94,188,709	94,212,651	both
*Xist*	X	down	103,460,373	103,483,233	both
*Slc25a53*	X	up	136,981,116	137,038,302	ChrX.3
*Kdm5c*	X	down	152,233,020	152,274,535	both
*Rps7-ps3*	X	down	152,909,505	152,910,155	ChrX.3
*Kdm5d*	Y	up	897,566	943,813	both
*Eif2s3y*	Y	up	1,010,543	1,028,847	both
*Uty*	Y	up	1,096,861	1,245,759	both
*Ddx3y*	Y	up	1,260,771	1,286,629	both

## Discussion

In this study, we leveraged wild-derived PWD mice compared with the classic laboratory strain, B6, together with the derivative B6.Chr^PWD^ consomic mouse panel, to study host genetic basis of susceptibility of severe IAV infection. While our study is certainly not the first to use forward genetics and quantitative trait locus (QTL) mapping in the mouse to address the role of host genetics, our approach is unique in that it: 1) used wild-derived mouse genetics, and 2) employed physical mapping in homozygous consomic mice, rather than mapping by association an intercross population, 3) intentionally considered sex as a covariate in the study design. In a physical mapping approach somewhat similar to ours, Schughart and colleagues used the (B6 x DBA/2J) BXD recombinant inbred strain panel to map susceptibility to PR8 H1N1 influenza, identifying a major QTL on Chr5 controlling weight loss and survival [45]. While it is possible that the effect seen in B6.Chr5^PWD^ mice in our study involves variants in same gene(s), this seems less likely because the DBA/2J rather than the B6 allele in the QTL on Chr5 was protective in the BXD study, whereas the PWD alleles on Chr5 resulted in enhanced susceptibility in our study. It is more likely that the wild-derived PWD alleles are distinct from the DBA/2J and B6 alleles present in the BXD panel. A similar study by Webby and colleagues also used BXD strains, but in the context of infection with highly pathogenic H5N1 IAV [46], where the results are also difficult to directly compare to ours, as none of the identified QTL overlapped with the PR8 H1N1 QTL study in BXD above, and thus may be unique to infection with highly pathogenic IAV. In another study, Vidal and colleagues used another recombinant inbred strain panel, the AcB/BcA (A/J x B6) panel to map susceptibility to infection with H3N2 IAV, identifying major loci on Chr2 and Chr17 [47]. Interestingly, unlike the other studies, this study used sex as a covariate and demonstrated distinct linkage patterns in males vs. females, with a significant effect of the Chr2 QTL only in females, and the Chr17 QTL only in males, in general agreement with our data suggesting distinct genotype-by-sex interactions in IAV susceptibility. The Chr17 QTL also overlapped with a minor QTL identified by Schughart and colleagues in the BXD PR8 infection model (see above), and it is consistent with our mapping study using B6.Chr17F^PWD^ and subconsomic B6.Chr17S^PWD^ strains, as it maps outside of the MHC locus. More recently, other investigators have also harnessed the power of wild-derived mouse genetics, in particular in the Collaborative Cross (CC) strain panel, which incorporates as founder strains B6 and other classic strains, as well as three wild-derived strains, including PWK [48], which is very closely related to PWD [30]. Using infection with PR8 H1N1 IAV in the incipient CC strains and CC founders, Ferris *et al*. identified several QTL controlling IAV susceptibility and associated phenotypes, including a QTL on Chr16 that contained *Mx1* [49]. The PWK allele at *Mx1* was associated with protection, which is fully consistent with our findings on complete IAV resistance in PWD and B6.Chr16^PWD^ mice, and the published findings that PWK mice are resistant to IAV infection [49, 50]. Our findings and those of others, however, do not confirm the sufficiency of *Mx1*^*PWD*^ to confer resistance to IAV, which could be shown in future gain of function experiments using transgenic complementation or *in vitro* transfection.

In contrast to our study, none of the above described IAV QTL studies identified significantly linked loci on X chromosome. This may stem at least in part from the failure to integrate sex into the study design to yield sufficient statistical power to detect differences in linkage across sexes. Interestingly, Lu, Schughart and colleagues recently performed a second QTL mapping study of PR8 H1N1 susceptibility in female BXD mice, augmented by lung transcriptomics and Bayesian network analyses, which identified a QTL on distal ChrX, with *Ace2* emerging as the lead candidate gene [51]. This QTL overlaps with our ChrX.3 locus, and thus may represent a shared genetic mechanism. While we did not observe differential expression of *Ace2* itself in the lung of B6.ChrX.3^PWD^ mice compare with B6 mice, this gene was shown to be downregulated after IAV infection [51], thus potentially masking any baseline differences.

Compared with B6, male but not female B6.ChrX.3^PWD^ mice exhibited elevated morbidity and mortality after IAV infection, associated with modestly elevated viral load and dysregulated inflammatory gene expression in the lung. The genetic mechanism underlying these phenotypes and the associated sex difference is unclear. Given the high potential for differential gene expression of ChrX-linked genes between males and females due to incomplete X inactivation [28], we speculate that differential genotype-dependent X-inactivation may be a driver of these phenotypes. In this scenario, specific allelic variants in the ChrX.3 locus may undergo differential X inactivation depending on the allele (B6 vs. PWD), leading to their differential expression in a sex-by-genotype dependent manner. While it may seem counterintuitive that the genotype effect in our study was seen in males and not females, it is quite possible that the original difference that existed between B6 males and females and was normalized by using sex-adjusted viral inoculum (**Fig 2**) is in part due to X inactivation in female B6 mice, and this inactivation is modulated by the ChrX.3^PWD^ locus. In support of this, we did identify several genes in the ChrX.3 locus that were differentially expressed in the lungs of ChrX.3^PWD^ mice in a sex-dependent manner (**Table 3**). Interestingly, this gene list included *Tlr13*, encoding a sensor of bacterial and viral RNA [44], which represents an intriguing gene candidate for future follow up studies. We identified several other X-linked genes that showed sex-biased expression in a genotype-dependent manner (**Table 4**), although their potential functional mechanism is unclear. Of these, *Slc25a53* is a curious positional candidate, representing an uncharacterized member of a family of mitochondrial transporters [52]. Interestingly, *Xist*, encoding the long non-coding RNA responsible for X inactivation in females, is also located on the distal end of ChrX, captured in the ChrX.3 locus. While this gene itself was not differentially expressed between ChrX.3^PWD^ and B6 mice, there are numerous polymorphisms in this gene distinguishing B6 and PWD that could have functional significance for X inactivation. An alternative non-mutually exclusive mechanism driving our ChrX-dependent phenotypes could be represented by the so-called intragenomic conflict between multi-copy genes on X and Y chromosomes [53, 54], which we have previously implicated in ChrY-dependent autoimmune phenotypes [55, 56]. Lastly, it is also possible that the sex-dependent effect of the ChrX.3^PWD^ locus is driven by the interaction of male and female sex hormones with genetic variants. Future experiments can address these intriguing possibilities.

Taken together, our study not only identifies novel host loci regulating susceptibility to severe IAV infection, but also demonstrates that sex differences in IAV severity are also regulated by host genotype. This has important considerations for future studies of both host genetic determinants and sex differences in human IAV infection, and it suggests that both variables need to be addressed in the study design. Further, our study reveals that natural genetic variation on ChrX can regulate susceptibility to IAV in a sex-dependent manner. Surprisingly few GWAS studies in humans have identified candidate genes on ChrX for any disease [57], despite the fact that this chromosome is large and carries many protein-coding genes, many of which have important immune-regulatory functions. In this regard, random X-inactivation in heterozygous individuals may represent a technical barrier [57], which is not an issue in our fully homozygous mouse model. Future studies will reveal whether X-linked genes in humans can regulate susceptibility to IAV, and whether this contributes to the well-documented sex differences in IAV severity.

## Supporting information

S1 File DEGs from RNAseq analysis. (**A**) DEGs between ChrX.3 females and B6 females. (**B**) DEGs between ChrX.3 males and B6 males. (**C**) DEGs between B6 males and B6 females. (**D**) DEGs between ChrX.3 males and ChrX.3 females. This is an Excel spreadsheet containing the full set of all genes in the analysis, in the with active DEG filters enabled, as indicated in each tab.
(XLSX)
